# Cancer of the Tongue

**Published:** 1895-03

**Authors:** E. W. Stevens

**Affiliations:** Philadelphia


### CANCER OF THE TONGUE.[1]

BY E. W. STEVENS, M.D., PHILADELPHIA.

From its structure and surroundings the tongue is peculiarly liable to be the seat of cancer. The disease is always of the squamous-celled epithelial type, scirrhus being rarely, if ever, found in this organ. Sarcoma of the tongue is rarely seen, but six cases being recorded in surgical literature.

Epithelial cancer is more frequently met with in the tongue than in any other organ or part of the body excepting the uterus. In 860 cases of epithelioma, collected from the Brompton Cancer Hospital case-book by Mr. Jessett ("Cancer of the Mouth," etc., London, 1892), the tongue was the seat of the disease in 190 cases, or at the rate of 22 per cent.; the uterus was affected in 222 cases, or at the rate of 25.8 per cent.; while the lips were affected in 160 cases, or at the rate of 18.6 per cent. The tongue also stands very high in the scale as compared with other organs affected by any form of cancer. At the Brompton Cancer Hospital, out of a total of 2227 cases of cancer occurring in patients during the ten years from 1882 to 1892, the tongue was the seat of the disease in 190 cases, or at the rate of 8.5 per cent. In 501 cases of carcinoma, collected at the Middlesex Hospital by Mr. Henry Morris, the tongue was the seat of the disease in 7.1 per cent. of cases. In Mr. Arthur Barker's 343 cases, at the University College Hospital, he found as high as 16.3 per cent. of cases of cancer of the tongue; while Sir James Paget, in 500 cases, found 6 per cent. Von Winiwater, in an analysis of 520 cases of cancer, found the tongue affected in 8.3 per cent. Dr. Sibley, on the other hand, in 520 cases, showed the tongue to be the seat of the disease in only 2.6 per cent. of cases.

Lingual cancer is extremely infrequent before thirty years of

---

[1] Read before the Academy of Stomatology, January 8, 1895.

age. A case has been recorded as early as the twenty-second year by Dr. Shepard, of Montreal (" Reference Hand-Book of the Medical Sciences"). In 58 cases collected by Mr. Whitehead, the oldest was seventy-six and the youngest thirty years of age. In the total of 133 cases collected by Dr. Gross, he found 82 were above fifty years and 51 under that age. The oldest was seventy-eight years of age and the youngest twenty-nine. In 190 cases analyzed at the Cancer Hospital by Mr. Jessett, the oldest patient was seventy-nine and the youngest thirty-two, the average age of the whole number being fifty-two years. The great majorty of cases occur between the ages of forty-five and sixty years.

Epithelial cancer of the tongue is much more frequent in men than in women. In a total of 485 cases collected from the tables of Wolfler, Rose, Paget, Barker, Clarke, Morris, Von Winiwater, and Jessett, 414 were males and 71 females.

Heredity does not appear to be an important factor in the production of this disease. In Mr. Whitehead's 104 cases, there was a history of cancer in the family in only six cases. Herr Von Winiwater states that inheritance is excluded in almost all his cases; while in those collected by Mr. Arthur Barker and Mr. Henry Morris, family taint was positive in but four cases. According to Mr. Barker, " It would appear as though the occurrence of cancer in the families of those who have the disease in the tongue was little more than a coincidence."

The question of the liability of smokers to cancer of the tongue is one to which a great deal of attention has been directed of late years. The disease is not more common among males who smoke than among those who do not smoke, while women who are addicted to the habit do not have cancer; and yet, as Mr. Batlin observes, it is not improbable that smoking does to some extent predispose to the disease. If smoking—and the same is true of spirit-drinking—causes sores or leucoma of the tongue, or chronic superficial glossitis, it predisposes the individual tongue in which these conditions are produced to carcinoma; but if smoking produces no appreciable effect upon the tongue, it cannot be said to predispose to cancer.

A large majority of cases of cancer of the tongue are preceded by an abnormal condition of its surface, which has been described under the various names of psoriasis, tylosis, ichthyosis, leukoplakia, keratosis, and leucoma. The term leucoma was first suggested by Mr. Jonathan Hutchinson, and is the only one usually employed. The designation chronic epithelial glossitis, proposed by

Besnier, would appear, however, to be a better one, as indicating its nature.

Leucoma is a chronic inflammation of the superficial mucous membrane of the tongue, which becomes deprived of its natural covering of papillæ, and instead of its normal red color it becomes bluish-white. The surface of the tongue becomes smooth and glazed, here and there marked by shallow furrows. The general appearance of leucoma is not unlike the effect produced by the application of nitrate of silver to the mucous membrane. The patient is not unusually much annoyed by the conditions present. Frequently he may be wholly unaware of their existence. In a patient under my care at the present time with leucoma of the tongue, which is known to have existed for six years, the dorsum looks as if it had received a thin coating of bluish-white paint, and is mapped with delicate furrows. At one spot, about six millimetres square, the tongue is uncovered, and is unnaturally red and somewhat raw. This patient has known for years that something was wrong with his tongue, but it gave him little trouble beyond a slight smarting at times, until about twelve months ago, when ulceration of the tongue ensued. When he first came under my observation, there was a fissured ulcer of the left anterior border of the tongue, with induration and thickening of the same side, sufficient to interfere with distinct articulation.

Leucoma of the tongue is never seen before twenty years of age. It may originate from syphilis or from an irritant, as rough and jagged teeth, smoking, or spirit-drinking; but however produced, it should always be regarded as a predisposing cause of cancer. The first appearance of carcinoma may vary within very wide limits. It may commence as a fissure or ulcer, a pimple or tubercle, a wart or warty growth, more rarely as a lump or nodule in the substance of the tongue. There is ample proof that many of these forms of disease in which cancer first appears are, in the first instance, and even for a long time after, simple non-cancerous affections, but which, being subject to irritation, gradually pass over into cancer.

This transition from a simple into a cancerous condition has been described, by Sir James Paget, as the pre-cancerous stage of cancer, and Hutchinson, Fournier, Butlin, and others, have emphasized the frequency with which non-cancerous lesions of the tongue undergo cancerous degeneration. Hutchinson has traced, on more than one occasion, the gradual formation of a cancer from a simple ulcer, or wart, and has shown how large a part the application of a caustic plays in the "breeding" of such cancers. Among the most common sources of irritation to which simple lesions of the tongue are subjected are irregular and rough teeth, smoking, spirit-drinking, and, above all, the application of a caustic, as the nitrate of silver. According to Butlin and Hutchinson, if there is one means more certain than another to transform a simple into a cancerous ulcer, it is the use of caustic, such as solid nitrate of silver, so commonly employed.

No clear line of demarcation, either clinically or histologically, can be drawn marking the transformation of a simple into a cancerous condition. The value of the microscope in this connection has probably been somewhat overestimated. Numerous cases have been reported where distinguished microscopists were wrong in their opinion, and what was presumed to be cancer turned out to be gumma. Moreover, a rapid spread of the disease is often seen after removal of a piece of the tongue for diagnostic purposes. When it is done the surgeon should operate at once, if necessary. Mr. Hutchinson recommends that the microscopic examination should be made after, and not before, removal of growth. According to Mr. Butlin, it is not necessary to remove a piece of the tongue in case of ulcer; scrapings from its surface can be used for examination under the microscope.

Any apparently simple lesion of the tongue which does not yield to treatment, after every source of irritation has been removed, should be excised at once, even in the absence of definite signs of cancer. Delay until the disease is fully developed is fatal, and takes away all chance of complete recovery from the patient.

When fully developed, lingual cancer, while widely different in different cases, presents a striking clinical picture. The increasing ulceration and induration, the pain often referred to the ear or temple, the salivation, the fixation of the tongue from infiltration so that it can no longer be protruded from the mouth, the enlargement of the lymphatic glands beneath the angle of the jaw, the gradually increasing difficulty of speech and swallowing, the not infrequent hemorrhages, and the progressive exhaustion mark the onward march of the disease.

The *diagnosis* of the affection at an early stage is most important, but of far greater importance is the recognition of the pre-cancerous stage. Curtis has lately shown that after operations for lingual cancer, in only 6.6 per cent. of cases does the disease not recur at the end of four years, and the proportion of cures after operation will always be comparatively few. The hope of surgery for the future in regard to lingual cancer lies in prevention rather than in cure (Butlin), and as the pre-cancerous conditions become more easily recognized by those who have the earliest opportunity of seeing them, and as the best methods of dealing with them and removing them at an early stage, if they do not yield to treatment, are more generally appreciated, so we may expect to see a decrease in the number of cases of lingual cancer.

The diseases with which cancer of the tongue is most likely to be confounded are syphilitic lumps and ulcers, tuberculous ulcers, warty tumors, and simple ulcers and fissures.

Simple ulcers and fissures heal rapidly without treatment when the source of irritation is removed, or under the use of simple applications. Should they not do so, cancer should be suspected, and they should be excised at once. There is one form of simple ulcer, the dyspeptic ulcer, which is not unfrequently met with, and which, while it improves under treatment, is prone to recur. It is superficial, and without induration, usually multiple, and appearing in successive crops. It is not likely to be mistaken for cancer.

The diagnosis between cancer and tuberculous ulcer of the tongue is often most difficult. Butlin quotes Nedopil as saying that the four tuberculous ulcers he had met with had all been cut out under the mistaken idea that they were cancer, and the diagnosis was only made after operation. Hajek has lately reported from Professor Schnitzler's clinic, at Vienna, a case of tubercular ulcer of the tongue, with concurrent tuberculosis of the larynx and lungs. A histological examination of a portion excised for the purpose failed to relieve the tubercle bacilli.

It is probable that some of the more brilliant cases reported, where life has been prolonged for many years after removal of the tongue for cancer, were tubercular and not cancerous. The mistake cannot be regarded as a serious one, the best treatment of tubercular ulcer being by excision. A less extensive operation, however, would, probably be indicated in most tubercular cases.

Tubercular ulcers are always secondary to deposits of tubercle in the lungs, and when a patient presents himself with an ulcer on the tip or side of the tongue, with no history of syphilis and with unmistakable signs of phthisis, we should suspect that such ulcer is tubercular.

Secondary syphilitic affections of the tongue would probably seldom or never be mistaken for cancer, and in chancre of the tongue the early glandular involvement and the development of the secondary symptoms will make clear the nature of the disease.

The points of resemblance between cancer of the tongue and the lesions of tertiary syphilis, however, are so numerous and striking that it is sometimes impossible to distinguish between them without the administration of antisyphilitic treatment, which in case of a shadow of doubt should always be employed. The difficulty which sometimes attends the diagnosis of lingual cancer from the ulcerations of tertiary syphilis is shown by the history of the following case:

Mr. T., aged thirty-six, merchant, was referred to me, by his dentist, for an intractable ulcer of the tongue of about four months' duration. Upon examination, the dorsum of the tongue presented the typical appearance of leucoma, with a fissured ulcer situated near the middle of the left border. The left half of the tongue was indurated and nearly twice the thickness of the right. For the past two weeks there had been an aching pain in the left ear. The ulcer was little, if any, painful, discharged very little, and presented a slightly raised base with the surrounding tissues somewhat angry. The glands beneath the angle of the jaw were apparently not enlarged. There was a family history of carcinoma, an uncle having died from cancer of the lip. Syphilis was denied absolutely. The patient smoked cigars and drank alcoholic liquor, but only moderately. The teeth were in excellent condition, and there was no history of injury to the tongue. My diagnosis of lingual cancer having been corroborated by three of the ablest surgeons in this city, to whom the case was referred for an opinion, an immediate operation for the removal of the tongue was strongly urged upon the patient. Owing, however, to some business transactions which required his immediate personal supervision, the operation was very reluctantly on my part postponed for a short period. Meantime a strict hygiene of the tongue was instituted, alcoholic liquors and smoking were given up, hot drinks, and irritating foods were avoided ; a mouth-wash, of a solution of borax and boracic acid, was used after eating, and without the slightest expectation of any benefit ensuing, the patient was placed upon antisyphilitic treatment.

In about two weeks' time the ulcer was healed and the induration and swelling of the tongue markedly diminished, and in ten weeks it was of normal size. The leucoma had not improved.

Two weeks of antisyphilitic treatment will usually be sufficient for a diagnosis, and should not be prolonged when improvement does not occur.

Finally, there is only one method of treatment of lingual cancer, removal by surgical operation. Operation not only effects a cure in a small number of cases, but it usually prolongs life and nearly always affords great relief. According to Mr. Barker, who has collected two hundred and eighteen cases of excision of the tongue for lingual cancer from the tables of Billroth, Whitehead, Rose, Kocher, and others, the average duration of the disease in cases not operated on was 11.7 months, and in those operated on nineteen months, a gain of 17.3 months. The elaborate tables of Wolfler correspond very nearly with those of Barker. Mr. Treves's estimate that the immediate mortality following excision of the tongue is now about ten per cent. may be considered nearly correct. The chief causes of death after operation are septic pneumonia, septicæmia, shock, and exhaustion.

